# Application of Transcranial Magnetic Stimulation with Electroencephalography in the Evaluation of Brain Function Changes after Stroke

**DOI:** 10.1155/2023/3051175

**Published:** 2023-05-24

**Authors:** Linhong Mo, Yiqiu Nie, Guiling Wan, Yingbin Zhang, Man Zhao, Jiaojiao Wu, Huiqi Wang, Qing Li, Aixian Liu

**Affiliations:** Neuro-Rehabilitation Center, Capital Medical University, Beijing Rehabilitation Hospital, Beijing 100144, China

## Abstract

**Objective:**

Based on transcranial magnetic stimulation (TMS) with electroencephalography technology, this study analyzed the rehabilitation mechanism of patients' motor function reconstruction and nerve remodeling after stroke. It revealed the function of the cerebral cortex network at a deeper level and established a set of prognostic marker evaluation indicators for the reconstruction of motor function after stroke.

**Methods:**

Twenty-one patients treated at the Beijing Rehabilitation Hospital of Capital Medical University because of ischemic stroke in the territory supplied by the middle cerebral artery were selected as the experimental group. Neurophysiological evaluation, motor function evaluation, and clinical evaluation were performed 30 and 180 d after the onset of ischemic stroke. In the control group, neurophysiological evaluation was also performed as a reference index to evaluate the changes in cortical patterns after stroke.

**Results:**

The brain topographic map showed the changes in energy or power spectral density (PSD) at 1,000 ms after stimulation as compared with before stimulation, but no difference was detected in these patients. The time-frequency analysis showed that when the left primary motor cortex (M1) area was stimulated using TMS, the PSD values of the left and right M1 and posterior occipital cortex areas produced an 8–40 Hz wave band in patients S1–S11. There was no significant energy change in patients S12–S16.

**Conclusions:**

For patients with different injury types, degrees of injury, and different onset periods, individualized intervention methods should be adopted. The evaluation methods should be as diverse as possible, and the rehabilitation effects of patients should be assessed from multiple perspectives to avoid the limitations of single factors. Possible mechanism: After brain injury, the nervous system can change its structure and function through different ways and maintain it for a certain period of time. This plasticity change will change with the course of the disease.

## 1. Introduction

World Health Organization (WHO) survey results demonstrate that the incidence of stroke in China ranks first in the world, twice higher than the United States. It is worth noting that stroke is no longer the “patient” of the elderly; more and more young people have joined them. In the two major categories of stroke, the incidence of ischemic stroke in China is 1.36 times the world average, and the incidence and mortality of hemorrhagic stroke are 2 times the world average. A population-based study showed that the prevalence of stroke in China continued to rise from 2013 to 2019 [1].

There are 2.5–3 million new stroke cases in China every year. With the improvement of diagnosis and treatment level, and rescue success rate in recent years, the survival rate of patients with stroke has increased. Although early treatment and intensive rehabilitation can significantly improve neurological impairment, more than 80% of stroke survivors and 40% of chronic patients [2] suffer from neurological dysfunction. Motor dysfunction is the most common symptom after stroke, affecting the independent living ability of patients [3] and increasing treatment costs and mortality.

Therefore, active and effective rehabilitation treatment to reduce disability is particularly important. The rehabilitation treatment of motor function after stroke has gradually developed from neural promotion technology to physical factor therapy. In recent years, motor imagery therapy [4], constraint-induced motion therapy [5], functional electrical stimulation [6], enhanced occupational autonomic activity [7], and repetitive transcranial magnetic stimulation (rTMS) [8] have become new methods of rehabilitation treatment. Based on the nonrenewable nature of nerve cells, the specific mechanism of motor function recovery after stroke is still unclear [9]. In general, the rehabilitation effect cannot currently meet expectations, and there is no consensus on the best rehabilitation method. What is the patient's potential rehabilitation capacity? Based on her/his clinical characteristics, what is the best rehabilitation strategy for this person? Which patients will benefit most from specific interventions? In view of these problems, it is urgent to identify and verify accurate neurophysiological markers related to persistent disability and successful stroke rehabilitation to improve the ability to predict long-term outcomes after stroke.

Neurophysiological changes of nerve cells occur spontaneously within days to months after brain injury. Functional magnetic resonance imaging (MRI), positron emission tomography (PET), electroencephalogram (EEG), and TMS are widely used to study these changes, which can analyze the correlation between local blood flow, metabolism, and neuronal discharge to detect cortical excitability [10]. Using different neuroimaging methods to study the special role of brain regions in functional recovery, understand the potential mechanism of spontaneous recovery after stroke [11, 12], and detect the target and key period of treatment intervention [13] has become the next research trend.

One of the four major technologies in brain science research is TMS. It uses electromagnetic induction to generate eddy current painlessly and generate action potentials in cortical neuron tissue [14, 15]. It is often used in the rehabilitation treatment of stroke, including limb, speech, and cognitive function rehabilitation [16–18]. In general, TMS acts on the primary motor cortex (M1) and motor evoked potential (MEP) and is recorded by surface electromyography of the target muscle. Monopulse TMS (TEP) has been used to study the difference of corticospinal tract integrity and cortical excitability in patients with stroke [19]. Steiner and Ward found that the presence or absence of MEP in paralyzed limbs can predict the response to motor skill training in patients with chronic stroke [20] and the functional outcome of acute stroke [21]. TMS-associated measures of cortical function and plasticity (such as the short latency afferent inhibition, the short-interval intracortical inhibition, and the cortical silent period) might add useful information in most cases of secondary dementia, especially in combination with suggestive clinical features and other diagnostic tests [22]. In the TMS assessment of stroke neurophysiology, dependence on M1 and the integrity of the peripheral corticospinal tract pathway limits the characterization of the underlying neural mechanisms of motor dysfunction, as many patients may not be able to induce measurable MEP, especially in the early stage of stroke [23]. Although TMS can be used to identify patients with significant recovery potential, especially when used in combination with MRI and standard clinical examinations [21], the lack of MEP records may limit the predictive role of TMS in functional recovery.

With the deepening of research, TMS can be combined with neuroimaging and neuroelectrophysiological techniques to monitor its stimulating effects on brain regions, such as functional MRI, PET, EEG, and TMS [24, 25]. These methods have their own advantages and disadvantages. The combination of TMS and EEG provides the possibility for noninvasive detection of brain excitability, connectivity, and transient state. In general, EEG is a low-cost, noninvasive functional neuroimaging technology that uses electrodes located on the scalp to quantify nerve potentials in the brain. Action potentials come from the mixed effect of inhibitory and excitatory postsynaptic potentials in neurons, which can be captured at the scalp and analyzed to help diagnose, prognose, and monitor treatment responses to various neurological disorders. Since functional changes are earlier than morphological and structural changes, EEG signals can be more intuitively and sensitively analyzed after digital processing to help diagnose, evaluate prognosis, and monitor the treatment response of neurological dysfunction [26]. The excellent time resolution (∼1 ms) combined with clinically feasible acquisition procedures makes EEG an attractive technique for assessing changes in brain activity after stroke.

Conversely, TMS induces the initial activation of the target region, followed by the triggering activity of signals transmitted by axons and synapses, resulting in late effects [27]. Studying the EEG signals of TMS (TMS–EEG) can provide a direct relationship between brain function and behavior and simultaneously evaluate a variety of neurophysiological processes, including cortical reactivity, local cortical excitation or inhibition, oscillatory activity, global brain connectivity [28], and neural plasticity. Unlike MEP, TMS–EEG can be used to evaluate the cortical response of almost any target brain to stimulation. It is only limited by the accessibility of the cortical target structure to TMS, is independent of spinal excitability, and is highly repeatable. Therefore, TMS–EEG provides the possibility of studying cortical interaction. The first EEG signal induced by TMS reflects the excitability of stimulating the cortex, i.e., the functional state, while the response of other cortical regions induced by stimulation can be characterized by the EEG connectivity index [29]. Additionally, TMS–EEG can bypass the subcortical structure and directly evaluate the cortical excitability and connectivity. Compared with the traditional intervention, TMS–EEG has advantages for patients with severe subcortical damage. In evaluating the neurophysiological mechanisms related to brain network reorganization after stroke, TMS–EEG has good research and application prospects [21, 30] and can be used as a neurophysiological marker for stroke recovery [31, 32]. It provides a unique insight into effective connectivity and the description of causal interactions between regions, including how well the activation of one region explains the activation of another [33]. Notably, this approach provides a causal model on the origins of activation in neural activity patterns and might define the functional strengths between regions. However, in clinical practice, there are relatively few systematic personalized TMS–EEG studies for patients with different types and degrees of injury, and there is no standard treatment system.

The main purpose of this study was to explore the rehabilitation mechanism of motor function reconstruction and neural remodeling in patients with stroke based on TMS–EEG technology to further reveal the function of the cerebral cortex network. Additional objectives were to explore the correlation between EEG characteristic parameters (event-related potential, brain connectivity, etc.) and clinical evaluation indexes of motor function, to explore the correlation between TMS-induced changes in cortical excitability and connectivity and motor function changes, and to establish a set of prognostic markers for motor function reconstruction after stroke based on TMS–EEG technology. To understand the changes in cortical patterns after stroke events, the TEP and frequency oscillations of spontaneous cortical activity were compared between patients and controls.

## 2. Materials and Methods

### 2.1. Study Design

From June 2019 to February 2020, 21 patients with ischemic stroke in Beijing Rehabilitation Hospital, Capital Medical University were selected as the experimental group, and seven healthy people were selected as the control group. Among them, five patients were unable to continue to cooperate due to lung infection and other reasons. The experimental group ended up with 16 patients. None of the 21 patients died at the time of discharge. Seven healthy people were compared as the control group.

The inclusion criteria were as follows: (1) all patients met the diagnostic key points of various cerebrovascular diseases in the 2019 Guidelines for Diagnosis and Treatment of Acute Ischemic Stroke of the Cerebrovascular Disease Group of the Neurological Branch of the Chinese Medical Association [34]; (2) all patients were confirmed by computed tomography or MRI imaging examination; (3) the patients had the first onset of stroke with stable vital signs and no obvious progress in neurological signs; (4) the cerebral infarction sites were the middle cerebral artery blood supply territories, including the thalamus, corpus callosum, and internal capsule; and (5) all patients signed an informed consent form. This study was conducted with approval from the Ethics Committee of Beijing Rehabilitation Hospital, Capital Medical University. (No. 2020bkky-022).

The exclusion criteria were as follows: (1) failure of vital organs or insufficiency of vital organs; (2) diseases with bleeding tendency; (3) unstable blood pressure control; (4) pacemaker and implantable defibrillator with other implantable devices in the body; (5) history of epilepsy; (6) any contraindications to TMS; or (7) failure to give informed consent. Seven healthy volunteers were selected as the control group, comprising two females and five males, aged from 42 to 54 years old (T1–T7).

### 2.2. Neurophysiological Assessment

The motor function assessment and clinical evaluation were performed at Days 30 and 180 after the onset of ischemic stroke.

### 2.3. Electroencephalogram and Transcranial Magnetic Stimulation with Electroencephalography Recording

The scalp EEG was recorded with TMS-compatible EEG equipment (Brain Amp 32 MR Plus, Brain Products GmbH, Munich, Germany). Using TMS-compatible Ag/AgCl electrodes mounted on elastic caps, the EEG was continuously obtained using 32 scalp positions located by the 10–20 international system. Additional electrodes were used for grounding and reference. The grounding electrode was located at the AFZ site, while the effective reference was located at the tip of the nose. The sampling rate of the EEG and electrooculogram (EOG) signals were 5 kHz and the band-pass filtering range was 0.1–1,000 Hz. The skin/electrode impedance was kept below 5kΩ. Horizontal and vertical eye movements were detected by recording EOG for offline signal processing to monitor the online behavior of participants and reject the offline trial through eye artifact.

### 2.4. Transcranial Magnetic Stimulation

The TEP was carried out by Magstim Super Rapid Stimulator (Magstim Ltd., Withland, Wales, UK). The stimulator was connected to a supercharger module and a standard digital 8 shape coil with an external winding diameter of 70 mm (Magstim Ltd.), which produced a maximum output of 2.2 T. To define resting motor threshold (RMT) [35], the coil was tangentially placed to the scalp, M1 on the left and right sides, with the handle pointing back and side 45° with the mid-sagittal axis of the participant's head, so that the current direction of the second stage was anteromedial. The stimulation started with the overthreshold intensity. The best stimulation site between the right and left first dorsal interosseous that elicits MEPs is called the motor hotspot, which is identified by placing the coil on the central sulcus and moving it to the scalp in steps of 0.5 cm on the left and right M1. The RMT was assessed as the lowest intensity required to evoke an MEP with a peak-to-peak amplitude ≥50 *μ*V in at least 5 out of 10 consecutive trials in the relaxed position. If MEP was not induced in the affected hemisphere (AH) at the maximum stimulator output (1% resolution), the intensity was set to 90% of the RMT of the unaffected hemisphere (UH). To target the left and right posterior parietal cortex (PPC), the coil was positioned on the tail of the inner parietal groove, roughly corresponding to P3 and P4, and oriented 15° from the midline, thus inducing current from the back to the front direction. For PPC, both hemispheres were symmetrical stimuli. High reliability was ensured during each recording. The coil location and the location on the hot spot were monitored online by using the SofTaxic neuronavigation system (EMS, Bologna, Italy) and a Polaris Vicra infrared thermal imager. To avoid the influence of auditory evoked potential brought by the TMS pulse sound on EEG signals, the subjects wore earplugs that played noise.

### 2.5. Exercise and Clinical Assessment

The Fugl–Meyer Assessment Scale (FMA) [36] was used to measure the recovery of limb motor function. The Barthel Index [37] comprehensively evaluated daily life, quality of life, and clinical rehabilitation ability.

### 2.6. Electroencephalogram Analysis

Two sets of EEG data obtained during the quiescent state were analyzed offline by MATLAB (MathWorks Inc., USA). Excessive drift, eye movement, blinking, and muscle activity were treated or excluded by independent component analysis. The energy or power spectral density (PSD) of different frequency bands was estimated by fast Fourier transform (10% Hanning window; frequency resolution: 1 Hz), which was divided into four frequency bands: delta (2–4 Hz), theta (4–8 Hz), alpha (8–12 Hz), and beta (12–30 Hz). The average PSD of each group of EEG data were calculated as the superposition average within the frequency band, and the total power of each frequency band of all channels was calculated to evaluate the EEG difference between the two groups.

The analysis to evaluate the cortical response to TMS in the time domain showed that the TEP impulse response induced by TMS was recorded from the first 100 ms to the last 500 ms at each evaluation time point and stimulation area. All periods were recorded for a period of 100 ms before baseline correction to TMS pulses.

The analysis to assess the cortical response to TMS in the time-frequency domain showed that, from different cortical regions of AH and UH, the oscillatory response to TMS pulses were detected from 1 s to 1 s later.

In the brain connectivity calculation, the directed transfer function was used to construct the upper triangular and lower triangular matrices [35] to represent the bidirectional connectivity between the afferent and efferent directions, and the diagonal elements were not included. The paired sample *t*-test was performed for the upper and lower triangular matrices of the state before and after the intervention, and the lead pairs with significant improvement were taken out to draw the brain connectivity map.

**Figure 1:**
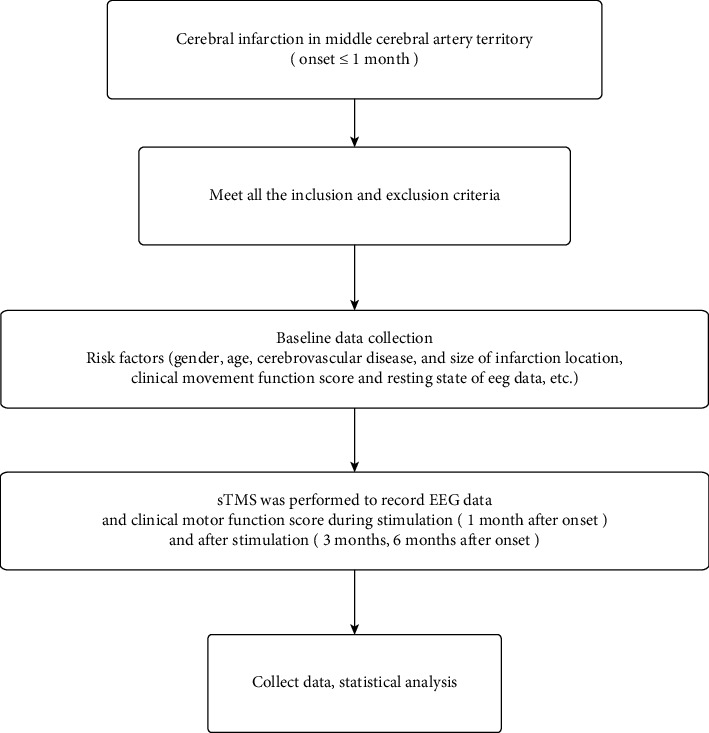
The flow chart of the study.

The research process is shown in Figure 1.

### 2.7. Statistical Analysis

The paired *t*-test was performed on the measurement data. The SPSS™ Statistics v23.0 software was used for statistical analysis. A value of *P* < 0.05 indicated a statistical significance.

## 3. Results

Among the 21 patients, 5 could not continue to cooperate due to pulmonary infection or other reasons. These left 16 patients in the experimental group (S1–S16) (Table 1). There are 13 males and 3 females in the experimental group, with an average age of 64. There were 3 males and 4 females in the healthy control group, with an average age of 51 years.

### 3.1. The Brain Electrical Signals and Electrical Energy

**Figure 2:**
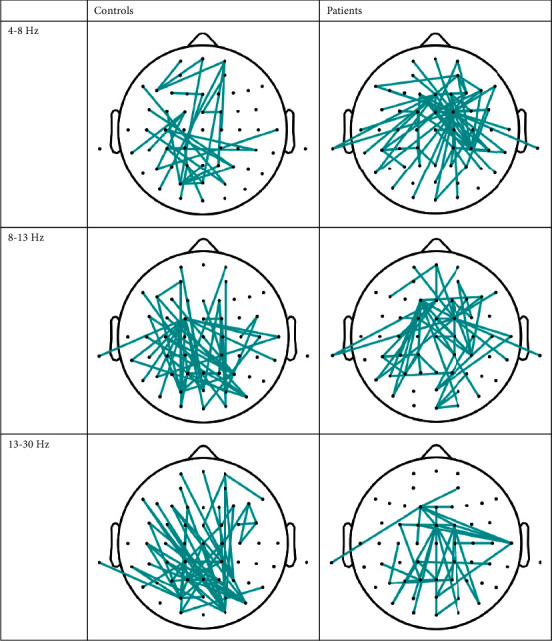
Comparison of the brain connectivity between the two groups.

After TMS, the brain electrical signals of healthy patients and patients with stroke changed, including brain electrical energy. After stimulation, the connectivity of the brain was significantly increased compared with before stimulation, which further proves that TMS can be used as a poststroke treatment (Figure 2). Rehabilitation treatment for stroke patients can improve limb dysfunction, improve the quality of life, and enhance the connectivity of the EEG.

### 3.2. Brain Topographic Map

The brain topographic map showed the changes of PSD at 1,000 ms after stimulation compared with 1,000 ms before stimulation. The energy changes of 8–40 Hz, the alpha band (8–13 Hz), and the beta band (13–30 Hz) were plotted, respectively. Satisfactory consistency could not be observed from the brain topographic map, and the differences in this experiment may not be measurable. (Appendix Figures 1A-1, 1A-2, 1B-1, and 1B-2).

### 3.3. Time-Frequency Analysis Chart

Appendix Figure 2 shows that the PSD values of 16 patients were calculated when the TMS of the left M1 area and the PSD of the left and right M1 area and the left and right posterior occipital cortex area under stimulation were changed, respectively. When the left M1 area was stimulated, the above four areas of S1–S11 produced an 8–40 Hz wave band as compared with the left and right M1 in patients S1–S7 and S9–S11. When the left M1 area was stimulated, the left M1 area energy increased higher than the right M1 area. Both sides were approximately equal for S8. There was no significant energy change in S12–S16.

### 3.4. Detailed Analysis for Patients

S1 (damage to the left hemisphere): The topographic map showed that after stimulating the left M1 area, the energy of the healthy right M1 dropped significantly. The time-frequency analysis showed that, when stimulating the left M1 area using TMS, the energy increase of the left M1 area was higher than that of the right M1 area, and the effect was higher than that of contralateral stimulation.

S2 (damage to the left hemisphere): The topographic map showed that after stimulating the left M1 area, the left hemisphere part of the energy increased, which had an exciting effect on the affected side. According to the time-frequency analysis diagram, when stimulating the left M1 area using TMS, the energy increase and the excitation effect in the left M1 area were higher than in the right M1 area.

S3 (damage to the right hemisphere): The topographic map showed that after stimulating the right M1 area, only the frontal parietal lobe had a small range of energy increase. The time-frequency analysis diagram indicated that when stimulating the right M1 area using TMS, the energy increase and the excitation effect in the right M1 area were higher than in the healthy left M1 area.

S4 (damage to the left hemisphere): The topographic map showed that after stimulating the left and right M1 areas, the energy of the occipital lobe area decreased, indicating an inhibitory effect but no obvious activation in the whole brain. The time-frequency analysis indicated when stimulating the left M1 area using TMS, the energy increase and the excitation effect in the left M1 area were higher than in the right M1 area.

S5 (damage to the left hemisphere): The topographic map showed that after stimulating the left M1 area, the frontal parietal lobe energy increased, indicating activation. The time-frequency analysis indicated that when stimulating the left M1 area using TMS, the left M1 area energy increased more and the energy excitation effect was higher than in the right M1 area.

S6 (damage to the right hemisphere): The topographic map showed that after stimulating the right M1 area, the right motor area showed a slight energy decrease in a small area, and the frontal and parietal area showed an increase in energy, but the scope of action was small. The time-frequency analysis indicated that when stimulating the right M1 area using TMS, the energy increase in the right M1 area was higher than in the left M1 area and the stimulation effect on the right side was higher than on the contralateral side.

S7 (damage to the left hemisphere): The topographic map showed that after stimulating the left M1 area, it showed an increase in energy, while the temporal lobe area showed a decrease in energy, indicating that TMS activated the patient's affected side's motor area. The time-frequency analysis indicated that when stimulating the left M1 area using TMS, the energy increase of the left M1 area was higher than in the right M1 area, meaning that the left-side energy excitation effect was high on the contralateral side of the excitation effect.

S8 (damage to the right hemisphere): The topographic map showed that after stimulating the right M1 area, the whole brain area showed a trend of energy reduction, indicating that there was an inhibition effect on the brain area. The time-frequency analysis indicated that when stimulating the right M1 area using TMS, the energy of the right M1 area increased more than that of the left M1 area, and the excitation effect on the right side was higher than that on the healthy side.

S9 (damage to the left hemisphere): The topographic map showed that after stimulating the left M1 area, the energy of the damaged left brain area increased significantly; that is, TMS activated the AH of the patient. The time-frequency analysis indicated that when stimulating the left M1 area using TMS, the energy increase and the excitation effect in the left M1 area were higher than in the right M1 area.

S10 (damage to the left hemisphere): The topographic map showed that after stimulating the left M1 area, the occipital lobe area decreased significantly. The time-frequency analysis indicated that when stimulating the left M1 area using TMS, the energy increase of the left M1 area was higher than in the right M1 area.

S11 (damage to the right hemisphere): The topographic map showed that after stimulating the right M1 area, only the occipital lobe showed a small range of energy increases. The time-frequency analysis indicated that when stimulating the right M1 area using TMS, the energy rise and the excitation effect in the right M1 area were higher than in the healthy left M1 area.

S12 (damage to the left hemisphere): The topographic map showed that after stimulating the left M1 area, the whole brain did not change significantly. The time-frequency analysis indicated that when stimulating the right M1 area using TMS, the energy decreased briefly after stimulation and then rose back to the baseline level.

S13 (damage to the left hemisphere): The topographic map showed that after stimulating the left/right M1 area, the contralateral inhibition effect was significant, but no significant change from the time-frequency analysis was observed.

S14 and S15 (damage to the right hemisphere): There was no significant change in the brain topographic map and time-frequency analysis map.

S16, damage to the right hemisphere: The topographic map showed that the energy of the occipital lobe was decreased slightly and it was inhibited, but no obvious change from the time-frequency analysis was observed.

**Figure 3:**
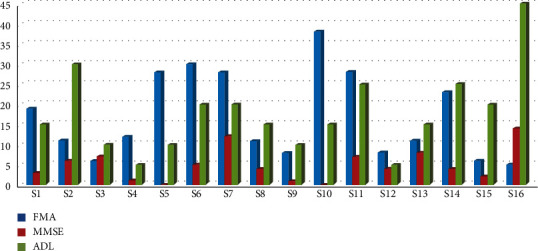
The distribution of clinical scale evaluation.

In summary (Figure 3):

Patients S1, S2, S7, and S9: Stimulation therapy using TMS should be applied to the affected motor area to enhance the activation of the affected side of the brain. At the same time, physical and occupational therapy should be provided to accelerate the nerve remodeling and motor function reconstruction of the affected side.

Patients S3, S5, S6, S10, and S11: After TMS treatment, the motor area of the injured side did not show obvious changes, and it was compensated by other brain regions. For these types of patients, the function of the compensatory brain area should be strengthened, combined with TMS, transcranial electrical stimulation, and other activation/inhibition treatment methods to activate the compensatory brain area. At the same time, the migration trend of the compensatory brain area should be observed, and the stimulation site should be adjusted accordingly.

Patients S4, S8, and S12–S16: TMS did not produce satisfactory energy activation phenomenon for such patients, so other treatments are suggested.

**Figure 4:**
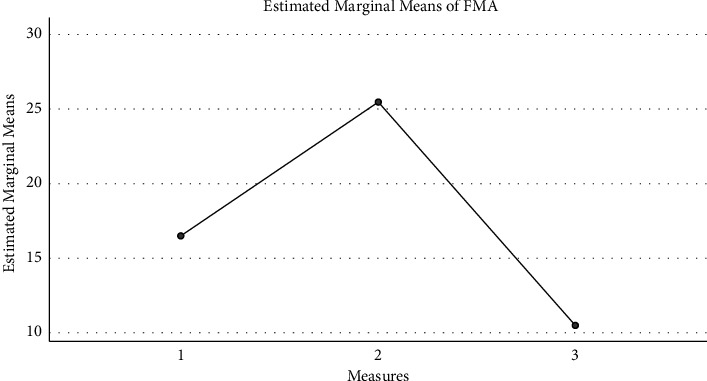
The FMA changes.

**Figure 5:**
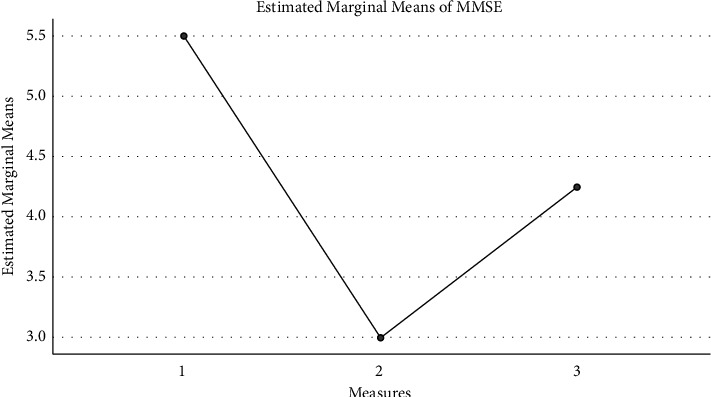
The MMSE changes.

**Figure 6:**
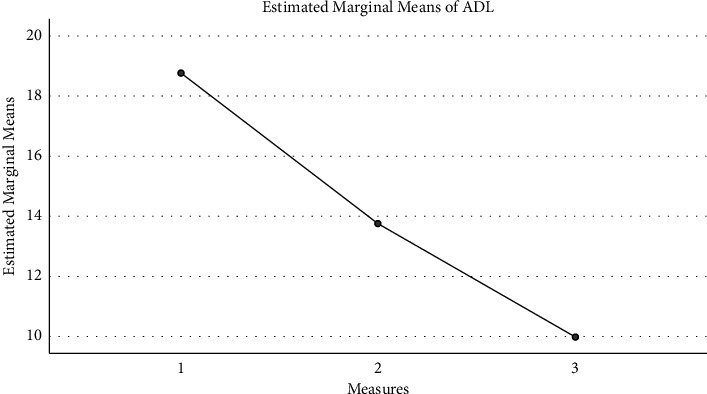
The ADL changes.

Based on the data of the topographic map and the time-frequency analysis, the patients were further divided into three groups: the in-situ expression group, the compensatory group, and the ineffective group.  Figure 4 shows the FMA changes: the in-situ expression group (16.5 ± 9.0), compensatory group (25.5 ± 13.7), and ineffective group (10.5 ± 1.7) intrabody effect test (*f* [2, 6] = 2.062, *P*=0.208) showed no significant difference among the three groups.  Figure 5 shows the Mini Mental State Examination (MMSE) changes: the in-situ expression group (5.5 ± 4.8), compensatory group (3.0 ± 3.6), and ineffective group (4.2 ± 2.9) intrabody effect test (*f* [2, 6] = 0.372, *P* = 0.704) showed no significant difference among the three groups.  Figure 6 shows the Activities of Daily Living (ADL) changes: the in-situ expression group (18.8 ± 8.5), compensatory group (13.8 ± 4.8), and ineffective group (10.0 ± 5.8) intrabody effect test (*f* [2, 6] = 1.657, *P*=0.267) showed no significant difference among the three groups.

## 4. Discussion

After stroke, various neurophysiological characteristics of the brain tissue change. The treatment strategy aims to increase neuronal plasticity, relearn processes and functional reorganization, form new neural networks and programs, improve functioning, and promote recovery. At present, the results of rTMS in the treatment of patients with stroke are different due to different parameters. Further research is needed to determine the best treatment method.

Lanza et al. found [2] TMS can be exploited as a noninvasive tool able to evaluate in vivo the cortical excitability, the propension to undergo neural plastic phenomena, and the underlying transmission pathways. The combination of TMS and EEG technology is highly praised for its simplicity and high time resolution advantages. It can provide a variety of neurophysiological processes at the same time and gradually shows its potential in evaluating the neurophysiological mechanism related to brain network reorganization after stroke and becoming a neurophysiological marker for functional recovery of stroke.

In this study, the EEG parameters of TMS in patients with stroke were evaluated, such as EEG power spectrum density, TEP, and brain network connectivity and their correlation with injury type, injury degree, and motor function prognosis were analyzed.

### 4.1. Evaluation of Whole Brain Electroencephalogram Power Spectral Density (Brain Topographic Map)

The lower frequency band of TMS and delta was selected to interfere with the motion of TMS and delta. Alpha (beta)-band energy can be used as an evaluation index. At present, the peak frequency of the alpha band is often used in current research, which varies among different populations. Quantitative EEG using spectrum content power spectrum analysis has been applied to stroke, and relevant studies have shown that there is a correlation between spectrum power and the level of disability after stroke [38].

In the resting state, the motor area energy of patients with stroke was lower than that of the whole brain. According to the brain topographic map, the power spectrum changes of 1,000 ms after stimulation were not observed in patients with stroke compared with those before stimulation. There was no consistent difference in the brain topographic maps of healthy people, but the energy change of the brain topographic maps in task state had good consistency. Compared with the active task, TMS cannot cause regular changes in the whole brain energy distribution, suggesting that the latter may depend on spontaneous neurophysiological activities or may be due to the differences in the location, degree, and duration of brain injury in patients with stroke. The stimulation site and frequency of TMS are relatively fixed, which needs to be further studied.

### 4.2. Electroencephalogram Time-Frequency Analysis Results

When stimulating the unilateral M1 area using TMS, the energy of the bilateral M1 area and the occipital 8–40 Hz band were significantly higher than that in the resting state. Most patients' stimulation side was higher than the contralateral side. The energy of S1–S11 increased significantly for 100 ms and then returned to the initial state.

These results showed that TMS influenced cortical electrophysiological activities. Some studies have found that the increase of alpha band energy is related to the improvement of clinical symptoms of stroke. Whether the energy increase of each band in this study is related to the recovery of motor function needs to be verified. If the electrophysiological changes under the action of TMS are related to the prognosis, TMS can be used as a means of rehabilitation, and its corresponding electrophysiological indicators can also predict functional prognosis.

As there were differences in bilateral energy increase after stimulation, if there are similar differences in healthy controls, it indicates that there may be attenuation of cortical activity transmission after TMS, so the energy recorded on the opposite side of stimulation is lower.

If the healthy control is bilateral symmetrical, it may be that the cortical functions such as excitability, connectivity, and other characteristics change during stroke, which may cause reorganization, movement, subcortical circuit activation/inhibition, or neurotransmitter imbalance. The recovery of motor function (Fugl–Meyer score difference) was compared between symmetrical and asymmetric patients with bilateral energy increase; if there was a statistical difference between the two, then this energy increase may reflect the state of intercortical activity after stroke, which can be used as an indicator to assess prognosis.

Different people have different responses to TMS stimulation. In this study, bilateral EEG energy increased after TMS treatment, which is inconsistent with some previous studies. What needs to be studied in the future is the following: is the hemisphere with high responsiveness related to the location, size, and onset time (time window) of TMS stimulation? Is the prognosis of patients with a good prognosis related to high energy on the same side of the lesion? Will the interhemispheric inhibition (stimulating one side of the M1 area can inhibit the contralateral M1) in a physiological state change after a stroke?

### 4.3. Brain Connectivity

The clinical outcome of stroke is related to local brain injury and to other parts of the connection. After a stroke, the coupling between hemispheres changes. Brain connectivity is considered as an index to study the mechanism of poststroke rehabilitation and evaluate the effect of some rehabilitation intervention measures. It can promote the rehabilitation process and produce adverse effects.

In this study, patients with stroke were found to have decreased brain connectivity compared with healthy controls in a resting state. After TMS stimulation, the brain connectivity of patients with stroke and healthy volunteers were significantly improved compared with forebrain stimulation, indicating that TMS can cause changes in the connectivity of the cerebral cortex and other parts but cannot quantitatively evaluate motor function and its correlation. Whether brain connectivity can be used as an effective guidance tool for TMS to treat stroke motor dysfunction remains to be studied.

### 4.4. Limitation of This Study

There are some limitations to our study, most notably is the small number and the rather heterogeneity in clinical features within the control group. The sample size is small, and it is impossible to judge the specific connection between the exact brain injury site and the neurophysiological characteristics of brain tissue, such as the cortical connectivity and TEP. In the future, it is necessary to explore the abnormal patterns and quantitative indicators of cortical activity at different times and in different parts after stroke and the specific relationship between abnormal patterns and neural function recovery, with an increased sample size. And the study of expanding the sample size is necessary to further determine the stability of the results. Due to the changes in cortical activity after stroke, the choice of TMS stimulation frequency and stimulation site needs to be personalized. Meanwhile, the changes in neurocortical plasticity at different stages of stroke should be considered and continuous observation is required. In addition, TMS itself generates noise, which may contaminate the activities induced by TMS. At present, there is no unified standard for the postprocessing of EEG signals, and multicenter validation is still needed to determine the reliability of TMS–EEG-related neurophysiological markers. In addition, studies have shown that transcranial direct (tDCS) and alternating current (tACS) stimulation have low electrical stimulation intensity on the scalp and have been widely used in this field [39], but there is a lack of comparative study of these three methods.

## 5. Conclusions

In healthy subjects and patients with stroke, TMS stimulation will enhance brain connectivity, and some brain regions will be activated, but different brains will react to it inconsistently, which seems to be related to the disease process and progress. In some patients, such changes in brain connectivity and activation of brain regions may be considered as adaptive responses to disease progression. This change of the brain changes with the time of stroke, its brain connectivity and activated brain areas will also change. The differentiation of TMS treatment effect seems to be consistent with it. Therefore, it is suggested that TMS treatment should be individualized rather than “one size fits all.” There were no statistically significant differences between the FMA, MMSE, and ADL indicators. For patients with different types of injury, degree of injury, and different onset period, individualized intervention measures should be adopted. The evaluation methods should be as diverse as possible, and the rehabilitation effect of patients should be evaluated from multiple angles to avoid the limitation of any single evaluation factor.

## Figures and Tables

**Table 1:** Information overview of the 16 patients.

Number	Gender	Age (yrs.)	Injury site	Paralysis side	Admission FMA	Discharge FMA	Admission ADL	Discharge ADL	Admission MMSE	Discharge MMSE
S1	Male	62	Cerebral infarction-left hemisphere	Right	53	72	15	30	12	15
S2	Male	55	Cerebral infarction-left hemisphere	Right	8	19	30	60	8	14
S3	Male	61	Cerebral infarction-right basal ganglia	Left	34	40	50	60	18	25
S4	Male	74	Cerebral infarction-left hemisphere(lacunar infarction)	Right	86	98	95	100	28	29
S5	Male	65	Cerebral infarction-left basal ganglia	Right	62	90	70	80	30	30
S6	Male	51	Cerebral infarction-right hemisphere	Left	8	38	30	50	15	20
S7	Male	46	Cerebral hemorrhage-left basal ganglia	Right	14	42	30	50	8	20
S8	Male	62	Cerebral infarction-right hemisphere	Left	8	19	5	20	12	16
S9	Female	77	Cerebral infarction-left hemisphere (lacunar infarction)	Right	0	8	10	20	5	6
S10	Male	76	Cerebral infarction (left basal ganglia, lateral ventricle)	Right	29	40	45	60	10	18
S11	Male	79	Cerebral infarction-right hemisphere	Left	70	76	5	25	24	26
S12	Male	78	Cerebral infarction-left basal ganglia	Right	84	92	90	95	18	22
S13	Male	32	Cerebral infarction-right hemisphere	Left	30	53	35	60	26	30
S14	Female	67	Cerebral infarction-right basal ganglia	Left	90	95	45	90	12	26
S15	Male	63	Cerebral infarction-left frontotemporal parietal lobe	Right	12	50	40	55	Aphasia	Aphasia
S16	Female	74	Cerebral infarction-right hemisphere	Left	28	56	25	50	22	29

## Data Availability

The data that support the findings of this study are available from the corresponding author upon reasonable request.

## Supplementary Materials

Supplementary Materials Appendix Figure 1. Changes of energy distribution in healthy people (A) and stroke patients (B) before and after intervention. A-1: Distribution diagram of energy change of healthy people before and after stimulation. A-2: Energy change distribution of healthy people after intervention compared with that before intervention (comparison of two resting states before and after stimulation). B-1: Energy change distribution of patients before and after stimulation. B-2: Energy change distribution of patients after intervention compared with that before intervention (comparison of two resting states before and after stimulation). Appendix Figure 2. Time frequency analysis of stroke patients before and after stimulation.
