# Supplementary material for: Application of Transcranial Magnetic Stimulation with Electroencephalography in the Evaluation of Brain Function Changes after Stroke
Source: Int J Clin Pract. 2023 May 24;2023:3051175. doi: 10.1155/2023/3051175 (PMC10232191; doi:10.1155/2023/3051175)
Supplement: Supplementary Materials — Appendix Figure 1. Changes of energy distribution in healthy people (A) and stroke patients (B) before and after intervention. A-1: Distribution diagram of energy change of healthy people before and after stimulation. A-2: Energy change distribution of healthy people after intervention compared with that before intervention (comparison of two resting states before and after stimulation). B-1: Energy change distribution of patients before and after stimulation. B-2: Energy change distribution of patients after intervention compared with that before intervention (comparison of two resting states before and after stimulation). Appendix Figure 2. Time frequency analysis of stroke patients before and after stimulation. [file 3051175.f1.docx]

| **Appendix Figure 1A-1 Distribution diagram of energy change of healthy people before and after stimulation** | | | |
| --- | --- | --- | --- |
|  |  | 8-13Hz | 13-30Hz |
| S1 | Left M1 | 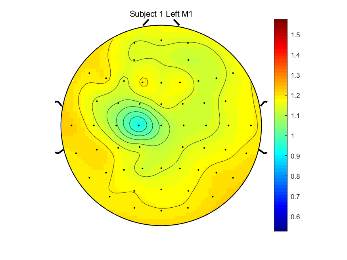 | 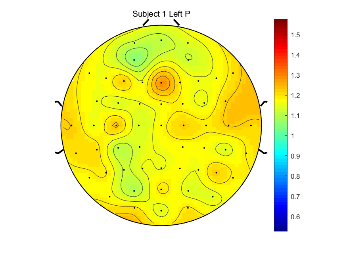 |
|  | Left PC | 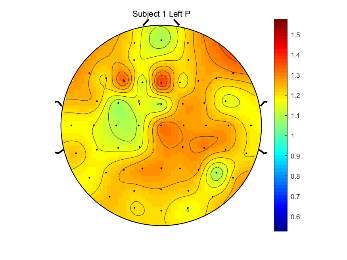 | 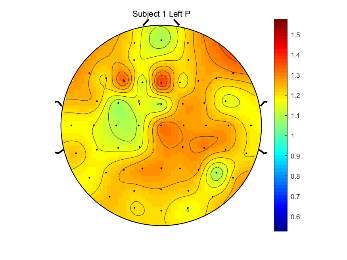 |
|  | Right MI | 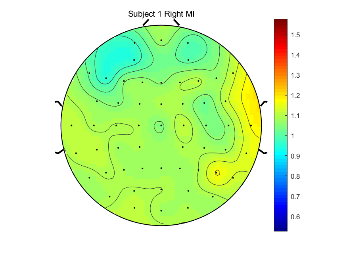 | 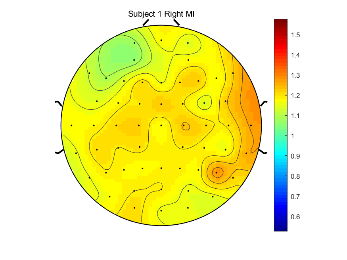 |
|  | Right PC | 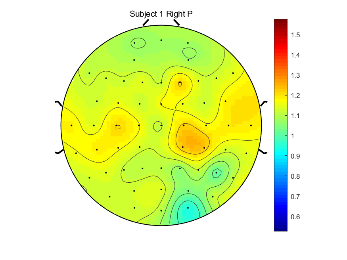 | 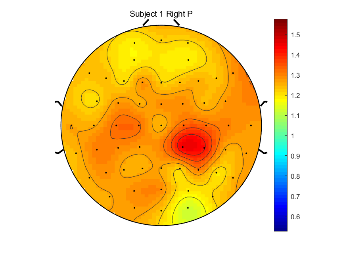 |
| S2 | Left M1 | 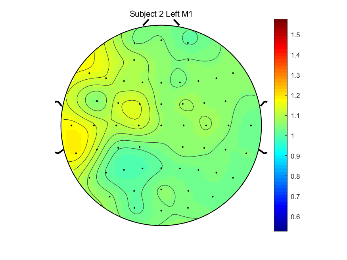 | 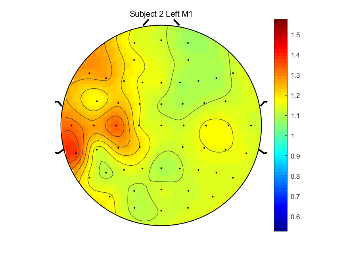 |
|  | Left PC | 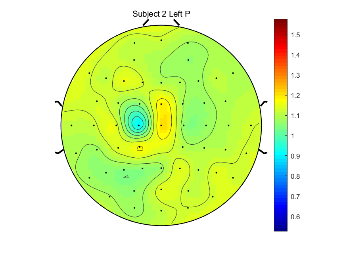 | 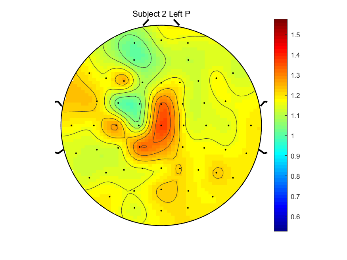 |
|  | Right MI | 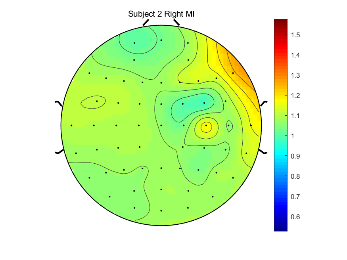 | 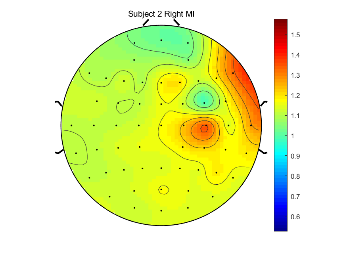 |
|  | Right PC | 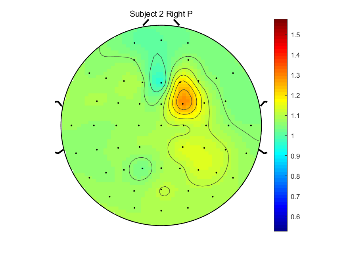 | 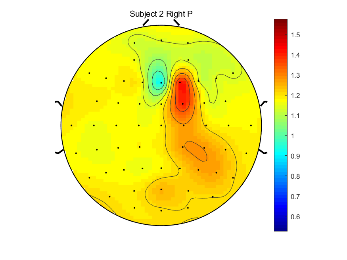 |
| S3 | Left M1 | 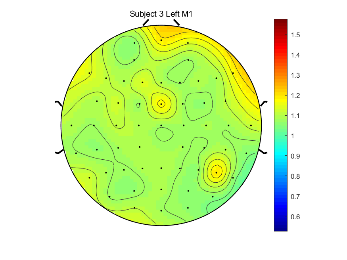 | 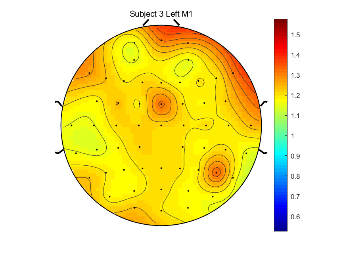 |
|  | Left PC | 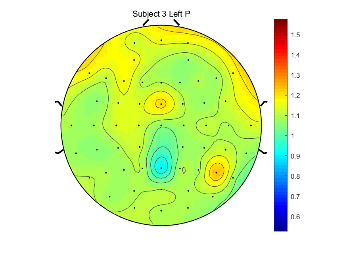 | 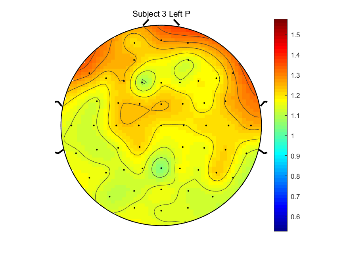 |
|  | Right MI | 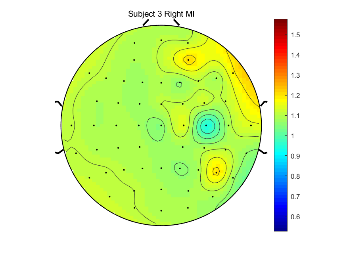 | 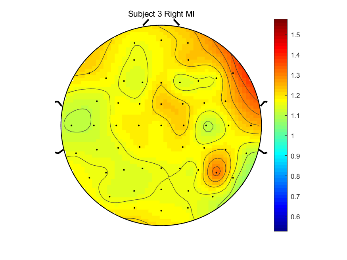 |
|  | Right PC | 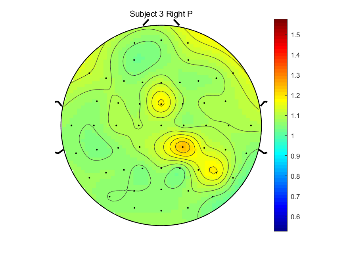 | 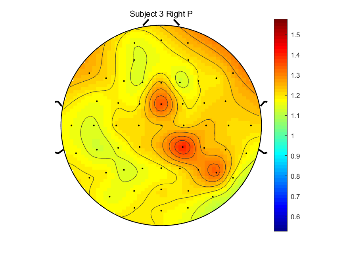 |
| S4 | Left M1 | 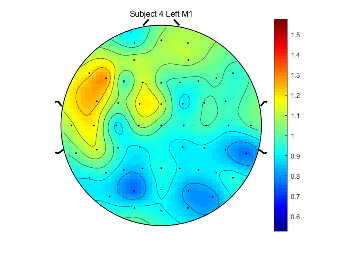 | 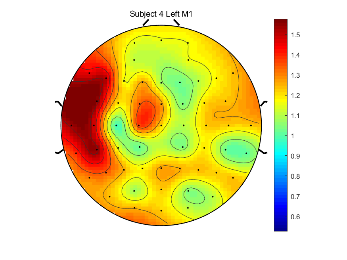 |
|  | Left PC | 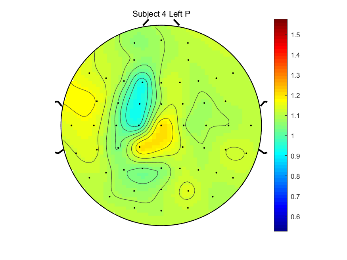 | 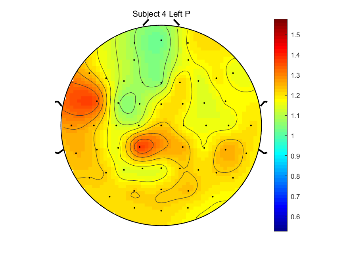 |
|  | Right MI | 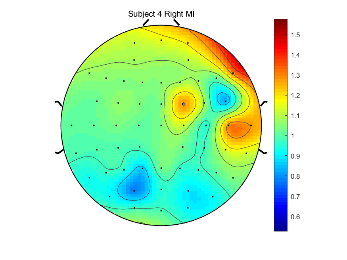 | 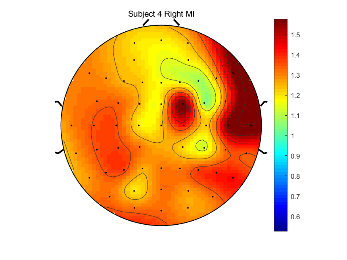 |
|  | Right PC | 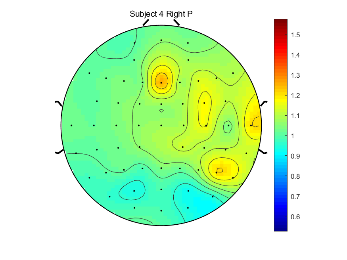 | 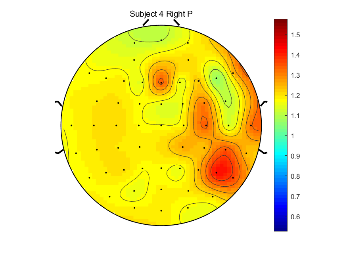 |
| S5 | Left M1 | 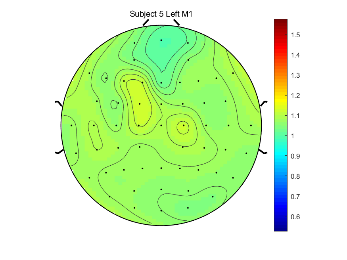 | 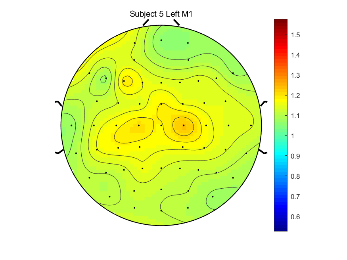 |
|  | Left PC | 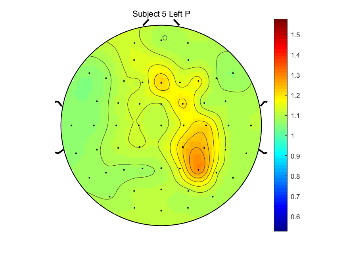 | 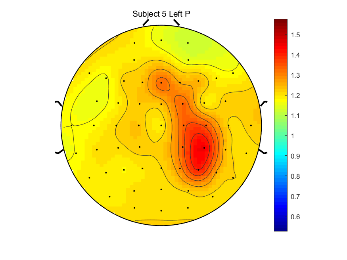 |
|  | Right MI | 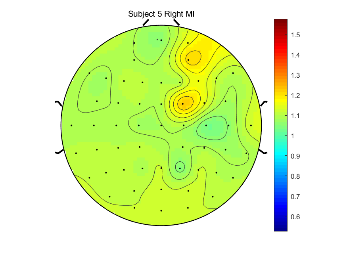 | 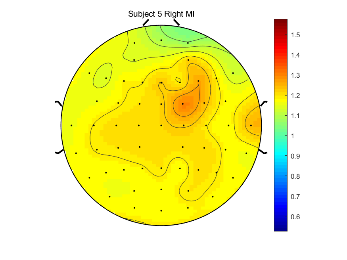 |
|  | Right PC | 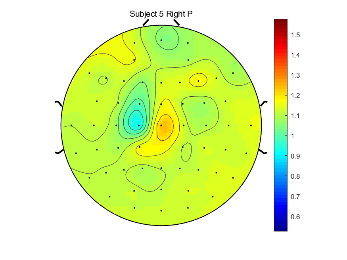 | 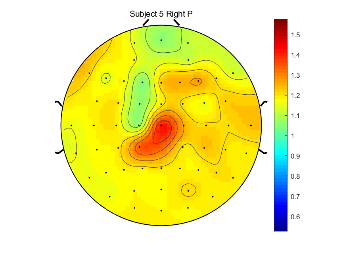 |
| S6 | Left M1 | 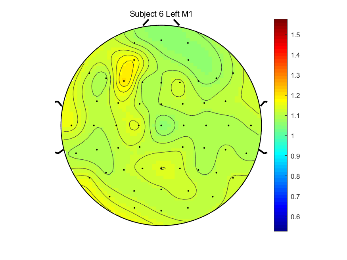 | 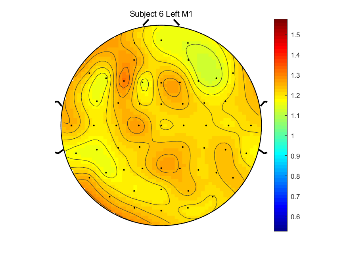 |
|  | Left PC | 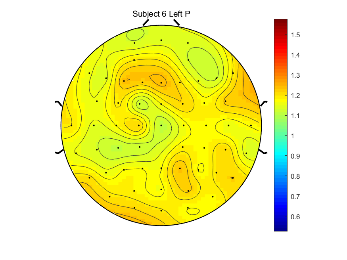 | 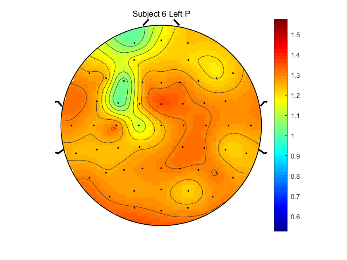 |
|  | Right MI | 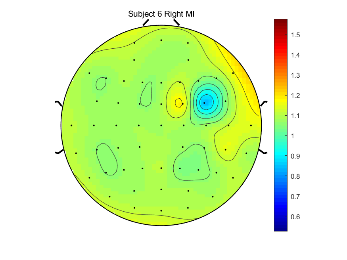 | 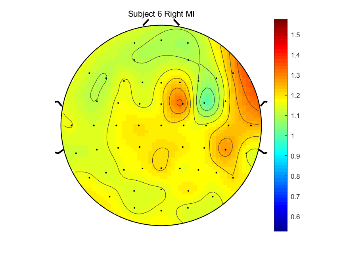 |
|  | Right PC | 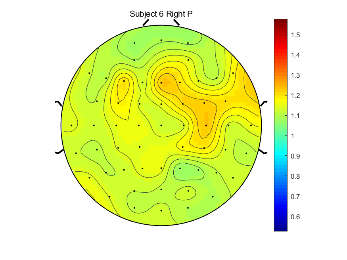 | 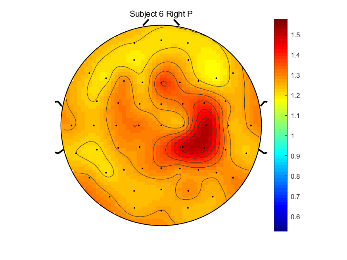 |
| S7 | Left M1 | 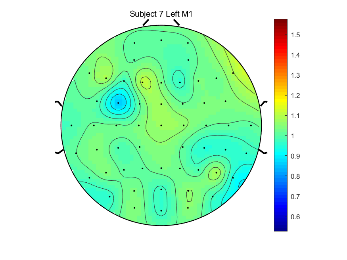 | 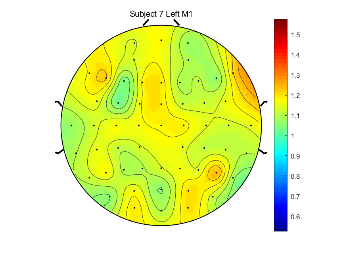 |
|  | Left PC | 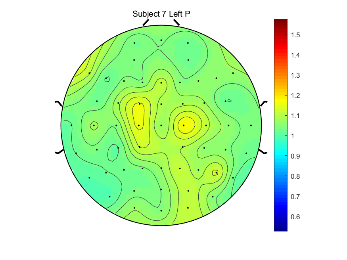 | 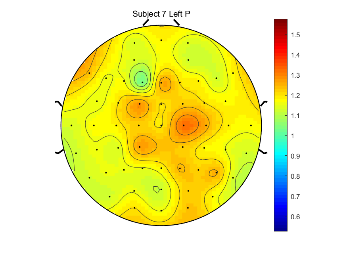 |
|  | Right MI | 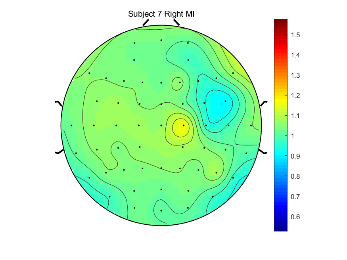 | 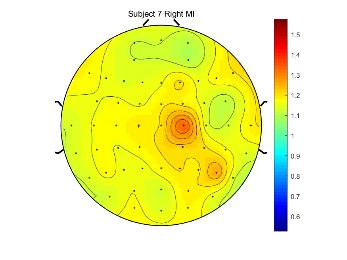 |
|  | Right PC | 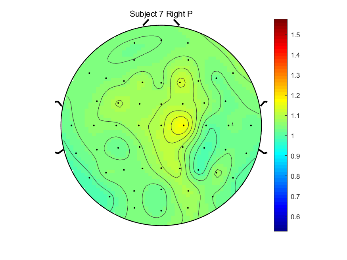 | 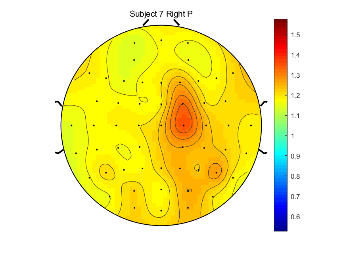 |

| **Appendix Figure 1A-2 Energy change distribution of healthy people after intervention compared with that before intervention (comparison of two resting states before and after stimulation)** | | | |
| --- | --- | --- | --- |
|  | 4-8Hz | 8-13Hz | 13-30Hz |
| S1 | **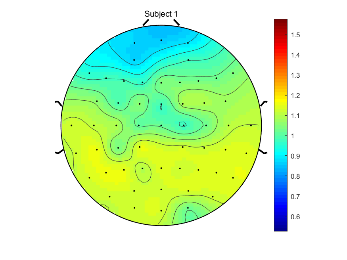** | 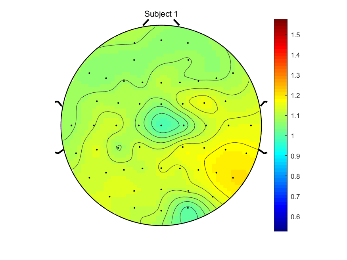 | 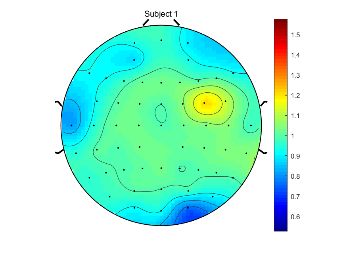 |
| S2 | 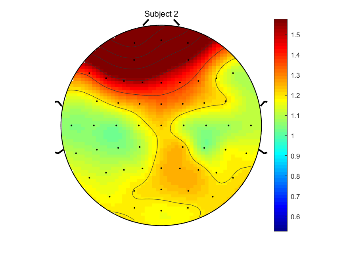 | 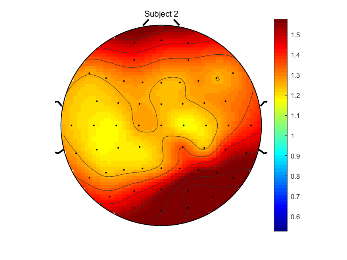 | 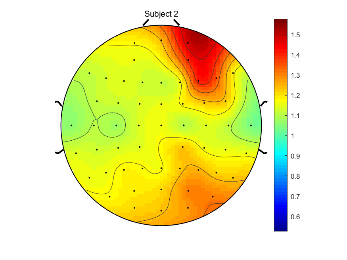 |
| S3 | 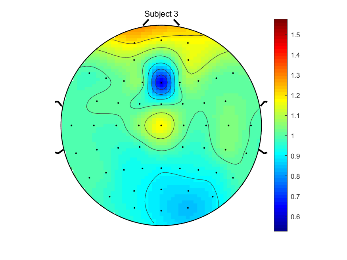 | 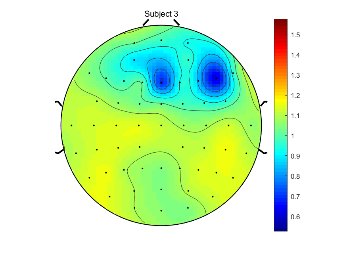 | 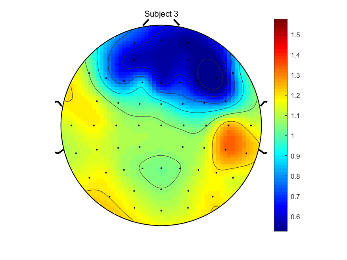 |
| S4 | 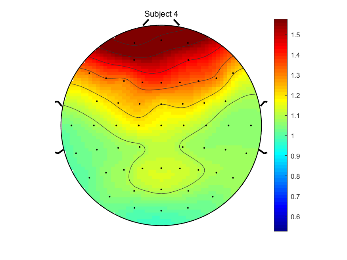 | 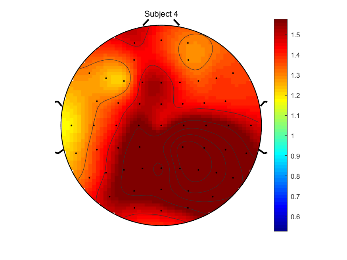 | 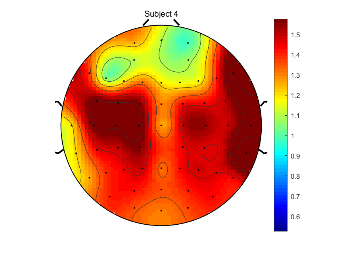 |
| S5 | 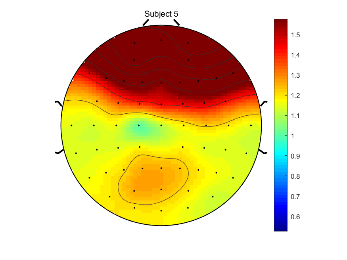 | 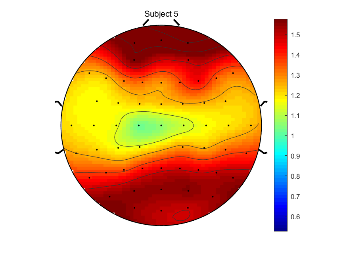 | 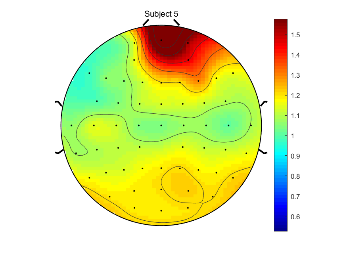 |
| S6 | 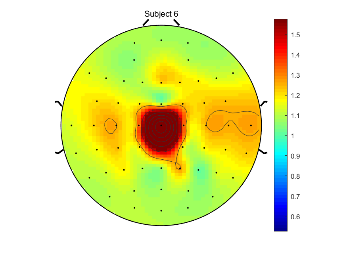 | 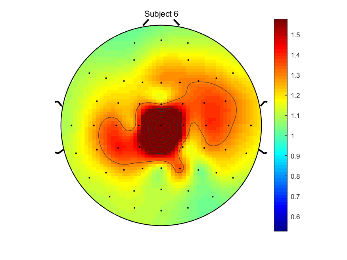 | 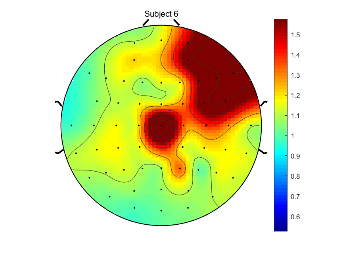 |
| S7 | 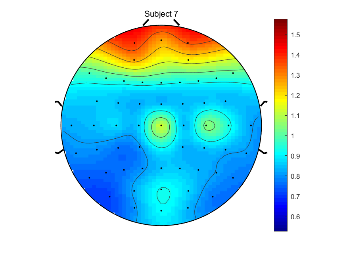 | 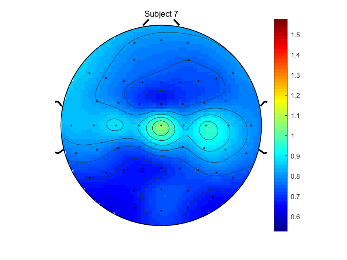 | 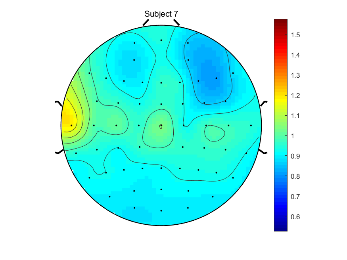 |

| **Appendix Figure1B-1 Energy change distribution of patients before and after stimulation** | | | |
| --- | --- | --- | --- |
|  |  | 8-13Hz | 13-30Hz |
| S1 | Left M1 | 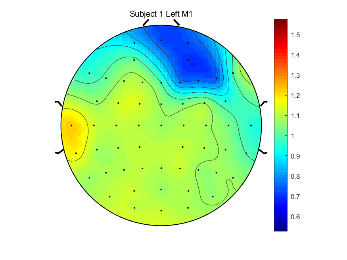 | 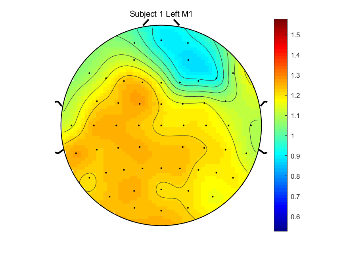 |
|  | Left PC | 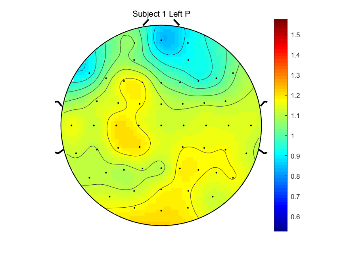 | 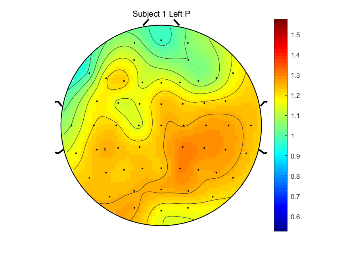 |
|  | Right MI | 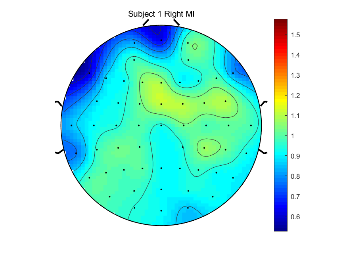 | 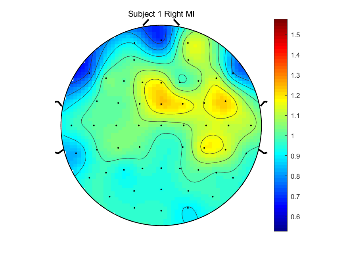 |
|  | Right PC | 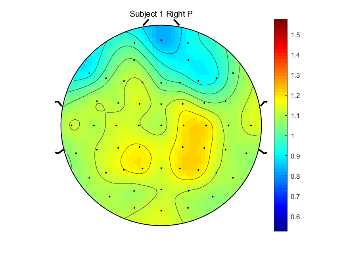 | 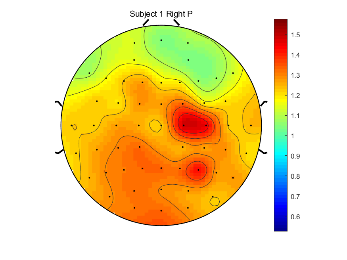 |
| S2 | Left M1 | 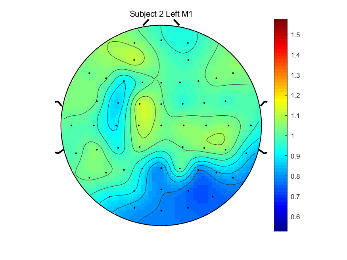 | 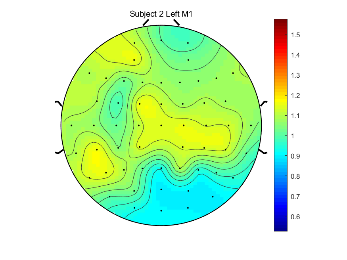 |
|  | Left PC | 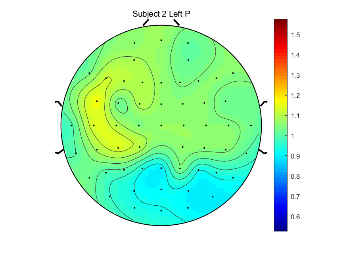 | 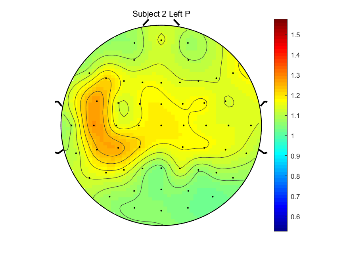 |
|  | Right MI | 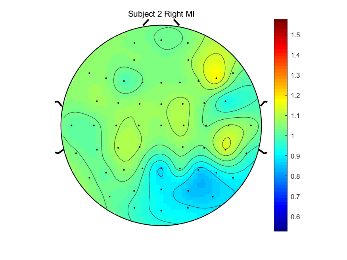 | 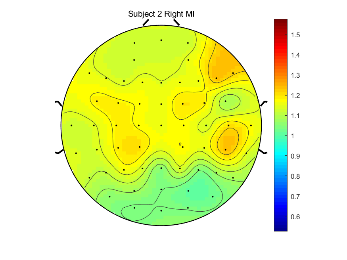 |
|  | Right PC | 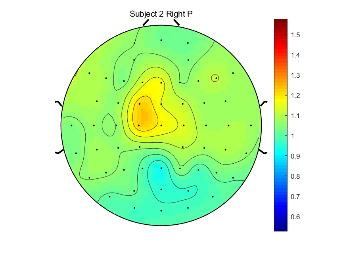 | 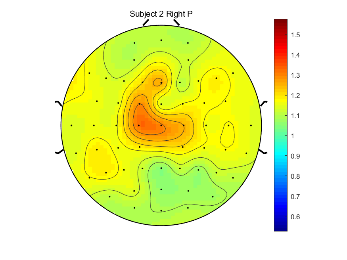 |
| S3 | Left M1 | 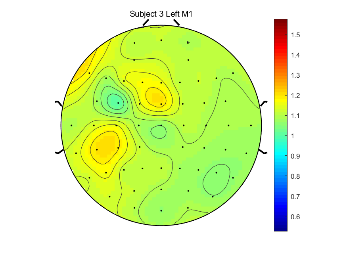 | 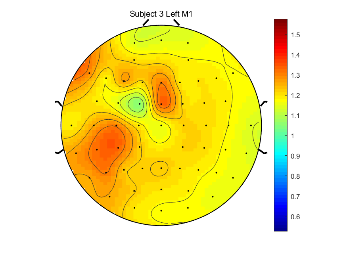 |
|  | Left PC | 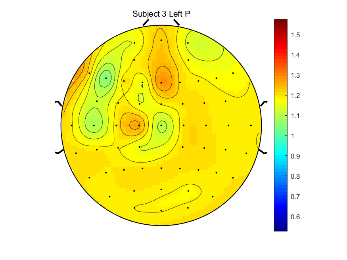 | 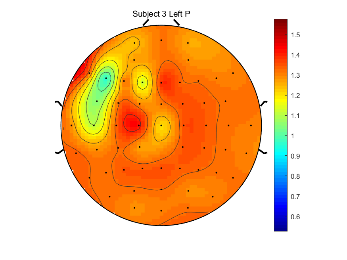 |
|  | Right MI | 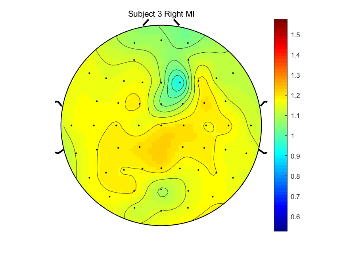 | 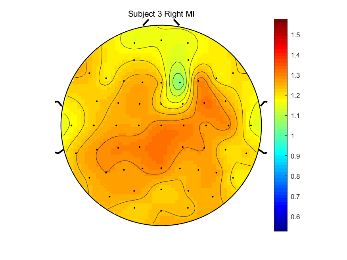 |
|  | Right PC | 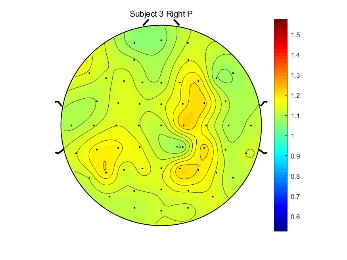 |  |
| S4 | Left M1 |  |  |
|  | Left PC |  |  |
|  | Right MI |  |  |
|  | Right PC |  |  |
| S5 | Left M1 |  |  |
|  | Left PC |  |  |
|  | Right MI |  |  |
|  | Right PC |  |  |
| S6 | Left M1 |  |  |
|  | Left PC |  |  |
|  | Right MI |  |  |
|  | Right PC |  |  |
| S7  徐海林 | Left M1 |  |  |
|  | Left PC |  |  |
|  | Right MI |  |  |
|  | Right PC |  |  |
| S8 | Left M1 |  |  |
|  | Left PC |  |  |
|  | Right MI |  |  |
|  | Right PC |  |  |
| S9 | Left M1 |  |  |
|  | Left PC |  |  |
|  | Right MI |  |  |
|  | Right PC |  |  |

| **Appendix Figure1B-2 Energy change distribution of patients after intervention compared with that before intervention (comparison of two resting states before and after stimulation)** | | | |
| --- | --- | --- | --- |
|  | 4-8Hz | 8-13Hz | 13-30Hz |
| S1 |  |  |  |
| S2 |  |  |  |
| S3 |  |  |  |
| S4 |  |  |  |
| S5 |  |  |  |
| S6 |  |  |  |
| S7 |  |  |  |
| S8 |  |  |  |
| S9 |  |  |  |

**Appendix Figure 2 Time frequency analysis of stroke patients before and after stimulation**

| **Stimulus Location: Left M1** | | | | |
| --- | --- | --- | --- | --- |
|  | Left MI | Right M1 | Left POC | Right POC |
| S1 |  |  |  |  |
| S2 |  |  |  |  |
| S3 |  |  |  |  |
| S4 |  |  |  |  |
| S5 |  |  |  |  |
| S6 |  |  |  |  |
| S7 |  |  |  |  |
| S8 |  |  |  |  |
| S9 |  |  |  |  |
| S10 |  |  |  |  |
| S11 |  |  |  |  |
| S12 |  |  |  |  |
| S13 |  |  |  |  |
| S14 |  |  |  |  |
| S15 |  |  |  |  |
| S16 |  |  |  |  |

Notes:

1. This figure calculated the EEG power spectral density values of 16 patients when TMS stimulated the left M1 area;

2. For each patient, the changes of power spectral density of left M1 area, right M1 area, left posterior occipital cortex area and right posterior occipital cortex area with time under stimulation were calculated;

3. When the left M1 region was stimulated by TMS, the above four regions in S1-S11 produced a significant increase of energy in the 8-40Hz band (alpha band, beta band, gamma band) for 100ms, and then fell back to the initial state;

4. By comparing the left M1 area and the right M1 area (by colorbar scale), it can be seen that S1-S7 and S9-S11, when TMS stimulates the left M1 area, the energy rise of the left M1 area is higher than that of the right M1 area; For S8, both sides are approximately equal.

5. No significant energy change was observed in S12-S16.

| **Stimulus Location: Right M1** | | | | |
| --- | --- | --- | --- | --- |
|  | Left MI | Right M1 | Left POC | Right POC |
| S1 |  |  |  |  |
| S2 |  |  |  |  |
| S3 |  |  |  |  |
| S4 |  |  |  |  |
| S5 |  |  |  |  |
| S6 |  |  |  |  |
| S7 |  |  |  |  |
| S8 |  |  |  |  |
| S9 |  |  |  |  |
| S10 |  |  |  |  |
| S11 |  |  |  |  |
| S12 |  |  |  |  |
| S13 |  |  |  |  |
| S14 |  |  |  |  |
| S15 |  |  |  |  |
| S16 |  |  |  |  |

Notes:

1. This figure calculated the EEG power spectral density values of 16 patients when TMS stimulated the right M1 area;

2. For each patient, the changes of power spectral density of left M1 area, right M1 area, left posterior occipital cortex area and right posterior occipital cortex area with time under stimulation were calculated;

3. When TMS stimulated the right M1 region, the above four regions in S1-S11 produced a significant increase of energy in the 8-40Hz band (alpha band, beta band, gamma band) for 100ms, and then fell back to the initial state;

4. Comparing left M1 area and right M1 area, it can be seen (by colorbar scale) : S1,S3,S4 and S6-S11, when TMS stimulates right M1 area, the energy rise of right M1 area is higher than that of left M1 area; For S2 and S5, the two sides are approximately equal.

5. S12 showed a transient decline in energy after stimulation and then increased back to the baseline level.

6. No significant energy change was observed in S13-S16.

| **Resting State** | | | | |
| --- | --- | --- | --- | --- |
|  | Left MI | Right M1 | Left POC | Right POC |
| S1 |  |  |  |  |
| S2 |  |  |  |  |
| S3 |  |  |  |  |
| S4 |  |  |  |  |
| S5 |  |  |  |  |
| S6 |  |  |  |  |
| S7 |  |  |  |  |
| S8 |  |  |  |  |
| S9 |  |  |  |  |
| S10 |  |  |  |  |
| S11 |  |  |  |  |
| S12 |  |  |  |  |
| S13 |  |  |  |  |
| S14 |  |  |  |  |
| S15 |  |  |  |  |
| S16 |  |  |  |  |

Notes:

1. This figure calculates the EEG power spectral density values of 16 patients within 4s in the resting state;

2. For each patient, the changes of power spectral density of left M1 area, right M1 area, left posterior occipital cortex area and right posterior occipital cortex area with time under stimulation were calculated;

3. No significant energy changes were found in the above four regions in the resting state under the 8-40Hz band (alpha band, beta band, gamma band), and the energy values were much lower than those induced by TMS stimulation.

Supplementary Description

Appendix Figure 1. Changes of energy distribution in healthy people (A) and stroke patients (B) before and after intervention.

A-1 Distribution diagram of energy change of healthy people before and after stimulation.

A-2 Energy change distribution of healthy people after intervention compared with that before intervention (comparison of two resting states before and after stimulation).

B-1 Energy change distribution of patients before and after stimulation.

B-2 Energy change distribution of patients after intervention compared with that before intervention (comparison of two resting states before and after stimulation).

Appendix Figure 2. Time frequency analysis of stroke patients before and after stimulation.
